# Precision Medicine for Hepatocellular Carcinoma: Clinical Perspective

**DOI:** 10.3390/jpm12020149

**Published:** 2022-01-24

**Authors:** Danijel Galun, Dragana Mijac, Aleksandar Filipovic, Aleksandar Bogdanovic, Marko Zivanovic, Dragan Masulovic

**Affiliations:** 1 School of Medicine, University of Belgrade, 11000 Belgrade, Serbia; draganamijac@gmail.com (D.M.); aleksandar.filipovic11@gmail.com (A.F.); aleksandarbogdanovic81@yahoo.com (A.B.); draganmasulovic@yahoo.com (D.M.); 2 HPB Unit, Clinic for Digestive Surgery, University Clinical Center of Serbia, 11000 Belgrade, Serbia; marko.zivanovic2005@gmail.com; 3 Clinic for Gastroenterology and Hepatology, University Clinical Center of Serbia, 11000 Belgrade, Serbia; 4 Center for Radiology and Magnetic Resonance Imaging, University Clinical Center of Serbia, 11000 Belgrade, Serbia

**Keywords:** hepatocellular carcinoma, precision medicine, targeted therapy, personalized treatment

## Abstract

Hepatocellular carcinoma (HCC) is one of the major malignant diseases worldwide, characterized by growing incidence and high mortality rates despite apparent improvements in surveillance programs, diagnostic and treatment procedures, molecular therapies, and numerous research initiatives. Most HCCs occur in patients with liver cirrhosis, and the competing mortality risks from the tumor and the cirrhosis should be considered. Presently, previously identified risk factors, such as hepatitis virus infection, hepatic inflammation and fibrosis, and metabolic syndrome, may be used as chemoprevention targets. The application of precision medicine for HCC management challenges the one-size-fits-all concept; moreover, patients should no longer be treated entirely according to the histology of their tumor but based on molecular targets specific to their tumor biology. Next-generation sequencing emphasizes HCC molecular heterogeneity and aids our comprehension of possible vulnerabilities that can be exploited. Moreover, genetic sequencing as part of a precision medicine concept may work as a promising tool for postoperative cancer monitoring. The use of genetic and epigenetic markers to identify therapeutic vulnerability could change the diagnosis and treatment of HCC, which so far was based on Barcelona clinic liver cancer (BCLC) staging. In daily clinical practice, the shift from a stage-oriented to a therapeutic-oriented approach is needed to direct the choice of HCC treatment toward the potentially most effective option on an individual basis. The important factor in precision medicine is the promotion of patient management based on the individual approach, knowing that the final decision must be approved by a multidisciplinary expert team.

## 1. Introduction

Hepatocellular carcinoma (HCC) is one of the major malignant diseases worldwide, characterized by unrestrainedly growing incidence, a low resectability rate, a high recurrence rate after curative-intent procedures, limited response to medical treatment, and, finally, a grave outcome. Today, more than 850,000 new cases are diagnosed annually, and it is expected that the burden of this disease might soon exceed an annual incidence of 1 million cases [1,2]. Moreover, HCC has one of the highest mortality rates among malignant tumors; the number of deaths recorded annually is close to the number of new cases diagnosed every year [3]. This proportion makes HCC an uncontrollable disease despite apparent improvements in surveillance programs, diagnostic and treatment procedures, molecular therapies, and numerous research initiatives. In the United States, the overall 5-year survival is less than 12%, making HCC the fastest rising cause of cancer-related deaths [4].

Disappointment with the results of HCC treatment is also reflected in the considerable differences between countries, which provide a disparate quality of healthcare regarding screening and surveillance programs, available treatment options, and reimbursement policies of state-funded health insurance.

Most HCCs occur in patients with underlying chronic liver disease and inflammation, i.e., liver cirrhosis. Therefore, clinicians should consider the competing mortality risks from the tumor and cirrhosis [5]. In western countries, chronic hepatitis C virus (HCV) infection; alcohol; and metabolic syndrome comprising obesity, diabetes, and nonalcoholic steatohepatitis (NASH) are all considered to be the leading risk factors [6]. In the eastern world, a background of chronic hepatitis B virus (HBV) infection is responsible for an increasing load of HCC cases [7,8].

In most centers worldwide, the treatment allocation is based on the modified Barcelona clinic liver cancer (BCLC) staging system endorsed by the European and American Association for the Study of the Liver [1,9]. The assessment of tumor burden, liver function, and general health status guides the selection of the best treatment modality for each patient with HCC [9]. However, no more than a third of HCC patients are candidates for curative-intent treatment options. For most patients, the diagnosis is established late, when limited treatment options are available, which results in poor treatment outcomes [9].

The application of precision medicine in HCC management challenges the one-size-fits-all concept; moreover, patients should no longer be treated entirely according to the histology of their tumor but based on molecular targets specific to their tumor biology [10,11]. Next-generation sequencing (NGS) emphasizes HCC molecular heterogeneity and aids our comprehension of possible vulnerabilities that can be exploited [12]. However, one of the major obstacles to implementing precision medicine for HCC patients is a reluctance to perform diagnostic biopsies [13]. One reason for this is that diagnostic imaging combined with α-fetoprotein is highly sensitive and specific for HCC. Another reason is the risk of tumor seeding via the biopsy tract [13,14]. Although biopsies offer limited histologic information that would impact clinical decision making, molecular information obtained by NGS may provide a critical breakthrough in the future treatment of HCC patients [12].

In this review, precision medicine for HCC is analyzed from the perspective of its clinical implication on risk factors and prevention, curative-intent procedures, and palliative treatment modalities.

## 2. Risk Factors and Prevention

Successful implementation of HCC-preventive strategies, including HCC screening, surveillance, and chemopreventive intervention, is not feasible without an individual, risk-based, and tailored approach [15]. However, the heterogeneity of HCC incidence based on etiology, patient ethnicity, and clinical implications hampers the effectiveness of preventive interventions. Presently, the precision medicine approach requires molecular information derived from specimens obtained by liver biopsy. Because of reluctance to perform diagnostic biopsies, a transition to less-invasive specimen types might help its wider applicability. Hopefully, in the future, more cost-effective and precise preventive interventions for patients at HCC risk will be feasible [15]. (Table 1 shows a comparison between liver and liquid biopsy). The potential of liquid biopsy in early diagnosis and evaluation of efficacy was highlighted by the 2019 Guideline for the Diagnosis and Treatment of Hepatocellular Carcinoma in China [16]. Several new serological molecular markers were described: circulating tumor cells (CTCs), circulating cell-free microRNA, and circulating tumor DNA (ctDNA). It was reported that a model for diagnosis of HCC using the levels of expression of seven plasma miRNAs can accurately diagnose early HCC (with a sensitivity of 86.1% and a specificity of 76.8%). The sensitivity of this model is about 30% higher than that of traditional markers [17,18]. Moreover, an HCC detection kit based on circulating miRNA is in clinical use in China [16].

Although liquid biopsy for ctDNA testing offers promising biomarkers for early cancer diagnosis, reported tumor measures demonstrate that when the fraction of tumor DNA falls below 0.01% of the total cfDNA, sampling of 10 mL of blood for ctDNA testing will most likely contain less than one cancer genome [19]. In such a low ctDNA level, an early cancer diagnosis is implausible [20].

Furthermore, ctDNA can be used as a tool for disease monitoring. In a study by Fujii and coworkers, ctDNA profiling was used for the treatment assessment in HCC patients treated with lenvatinib [21]. The study showed that somatic alterations could be detected in most advanced HCC patients by monitoring the level changes of variant allele frequencies (VAFs) in ctDNA. The reduction in VAFs was associated with longer progression-free survival. Moreover, the specificity and sensitivity of the reduction in mean VAF for predicting partial response were higher compared to that of serum AFP levels (0.67 and 1.0 vs. 0.10 and 0.93) [21].

Currently, HCC-preventive interventions are implemented on several levels. Primary prevention works with cancer-predisposing factors implementing vaccination programs, lifestyle modifications, or environmental interventions focusing on an etiology. Secondary prevention is related to patients exposed to already established etiological agents focusing on early detection and chemoprevention of HCC occurrence. Tertiary prevention focuses on reducing recurrences after the radical HCC treatment [22].

Despite cancer chemoprevention measures, limited progress was achieved in recent years, mainly because of the complexity of human carcinogenesis [23]. Several other obstacles limiting chemoprevention development are: (1) the lack of optimal animal models; (2) difficult access to liver specimens; (3) the lack of optimal biomarkers; and (4) ambiguity about the presence of informative biomolecules in the circulation that can be utilized by liquid biopsy [24,25,26].

According to current guidelines, regular biannual HCC screening is recommended [1,27]. At this stage, already identified risk factors—such as hepatitis virus infection, hepatic inflammation and fibrosis, and metabolic syndrome—may work as chemoprevention targets [15].

## 3. Hepatitis B

In the eastern world, chronic HBV infection is a dominant etiology, although HBV-associated HCC is declining [28]. Different from HCV infection, HBV DNA integration into the host genome causes direct cis/trans activation of oncogenic signals and carcinogenesis without requiring a fibrotic tissue microenvironment [29]. It was already reported that several predisposing factors, such as serum HBV DNA levels, certain HBV strains (e.g., genotype C in Asian and genotype F in Alaskan), and mutations in the HBV genome (e.g., precore and basal core promoter regions), are associated with increased HCC risk [29,30,31].

The established primary HCC prevention measure is a universal HBV vaccination reducing neonatal HBV vertical transmission [32,33]. The secondary prevention involves antiviral therapy. A meta-analysis of 2082 patients employing interferon-based regimens showed a reduction in the occurrence of cirrhosis and HCC development (RRs, 0.65 and 0.59, respectively) [34]. The use of nucleotide analogs (NAs) further reduced HCC incidence from 7.4% to 3.9% and from 13.3% to 1.1% in two trials on 651 and 2795 patients, respectively [35,36,37]. However, the impact of newer-generation, first-line NAs, entecavir and tenofovir, is ambiguous because studies from Asia reported an HCC risk reduction of 30% in cirrhotic and 80% in noncirrhotic patients, whereas evidence from the western world is still limited [38,39,40]. Regarding tertiary prevention (the use of antiviral therapy after the curative-intent liver resection or thermal ablation), a meta-analysis of 6350 patients reported lower recurrence-free survival (HR, 0.66) with the use of NAs [41]. The same finding is reported in another more recent trial with 200 patients (HR, 0.65) [42]. However, even if viral replication is attenuated by the NAs treatment, HCC risk is still present. It was confirmed that even if the HBV DNA level is consistently < 2000 IU/mL as a result of NAs treatment, the HCC incidence was significantly higher than the HCC incidence in inactive chronic hepatitis B patients, indicating that cancer risk is not completely eliminated by current antiviral therapies [43].

## 4. Hepatitis C

Despite evident progress in treating chronic HCV infection, the incidence and mortality of HCV-related HCC continue to rise in specific populations [44]. However, HCV clearance achieved by antiviral therapies with a sustained virologic response (SVR) is associated with reduced HCC incidence [45]. It was confirmed that interferon-based regimens failed to demonstrate any reduction in HCC occurrence because of the low treatment uptake (1–3% annually) and a modest SVR rate (50%) [46]. Although direct-acting antivirals (DAAs) demonstrated improved SVR, HCC incidence is predicted to further increase for several reasons: (1) low treatment uptake mainly related to high DAA costs and disparate reimbursement policies and treatment programs worldwide; (2) a high proportion of undiagnosed HCV infection; and (3) maintenance of the patient load from high-risk populations (inmates and injecting drug users/approximately 3–4 million new infections each year) [47,48,49,50]. Altogether, they result in a high disease burden that will continue into the near future even in developed countries [48,50,51].

Development of the primary prevention measure, i.e., a prophylactic HCV vaccine, is a challenging task because of the high viral genetic variability. Therefore, alternative strategies targeting the host genes/proteins are being considered [52,53]. Secondary and tertiary chemoprevention could be achieved by antiviral therapies in the knowledge that DAAs are better tolerated than interferon-based regimens in compensated and decompensated cirrhotic patients [54,55]. Nevertheless, recent studies reported a comparable level of reduction in HCC incidence between interferon- and DAA-induced SVR (HR, 0.28–0.29) [55,56]. Experimental studies indicate DAAs are associated with specific modulations of host immunity, such as reactivation of coinfected viruses; remission of follicular lymphoma; and restored function of HCV-specific CD8+ T cells, memory T cells, and normalized NK cells [45]. Therefore, the clinical utility of DAAs as part of the HCC chemoprevention strategy still requires elucidation.

## 5. Alcohol

Alcohol abuse is still one of the major HCC etiologies, mostly in northern Europe, although decreased alcohol-associated HCC mortality is registered in some countries [57]. The association between alcohol abuse and HCC risk is dose-dependent, as confirmed by a recent meta-analysis [58]. Hepatocarcinogenesis in patients with excessive alcohol consumption is mediated by acetaldehyde (ethanol metabolite), oxidative stress, and DNA damage; altogether, they create a carcinogenic microenvironment [59,60,61]. Although the extent of risk reduction by abstinence is not yet established, a meta-analysis demonstrated a reduced HCC risk of 6–7% annually [62].

## 6. Metabolic Syndrome

A metabolic syndrome comprising obesity, diabetes, and NASH is one of the major causes of the increasing incidence of HCC. The magnitude of the problem is reflected by the fact that a quarter of the global population is affected by nonalcoholic fatty liver disease (NAFLD), and among biopsied NAFLD patients, nearly 60% have nonalcoholic steatohepatitis (NASH) [63]. Obesity and type 2 diabetes are independent risk factors for HCC, and a high body-mass index (BMI) was significantly associated with liver cancer risk, according to recent research [64]. The results of a recent meta-analysis indicate that diabetes was associated with increased HCC risk (OR, 2.5 and HR, 2.5, respectively) [65]. Interestingly, the absence of cirrhosis is more frequently associated with HCC in NAFLD than other etiologies, suggesting that NAFLD-specific mechanisms of carcinogenesis are not necessarily dependent on hepatic fibrosis [66]. Metabolic proinflammatory cytokines and adipokines and their interference in obesity-related hepatocarcinogenesis were well established by several studies [67,68,69].

Observational studies indicate that lifestyle interventions may be used as *secondary* prevention measures for metabolic-syndrome-associated HCC. A meta-analysis involving nearly 1,300,000 participants suggested that increased consumption of vegetables may reduce HCC risk [70]. In another study that involved more than 420,000 individuals, higher physical activity was associated with reduced HCC risk [71].

## 7. Curative-Intent Procedures

According to the BCLC guidelines, curative-intent treatment modalities are indicated for early-stage HCC patients, and among them, liver resection and thermal ablation are the most commonly used procedures in both developed and developing countries. Strict adherence to the BCLC staging system limits surgical resection to HCC patients with single tumor <2 cm, Child–Pugh class A, performance status 0 without portal hypertension, and normal bilirubin level. However, the worldwide clinical practice of liver surgery for HCC is expanded to selected patients from the BCLC intermediate and even advanced stages. In many developing countries, screening and surveillance programs are lacking, and therefore, most patients are diagnosed in an advanced stage of the disease when surgical resection is still feasible [72,73,74].

Nevertheless, the results of liver resection are compromised by the recurrence of the disease, with the reported 5-year recurrence rate up to 74.2% [75]. Genetic sequencing as part of the precision medicine concept is a promising tool for post-operative cancer monitoring. In that perspective, ALB1 mutation was observed in the recurrent tumor tissue. Importantly, the study findings indicate that the time that the mutation can be detected in ctDNA is associated with the time when HCC relapses [76]. Cai and coworkers identified a mutation, HCK p.V174M, that was found in the recurrent and metastatic HCC, which was diminished after surgery but increased rapidly in the setting of HCC recurrence [77]. Another study confirmed that the IL-28B (rs8099917) TT genotype was associated with HCC recurrence [78]. The profile of circulating tumor DNA in blood samples of HCC patients indicates the tumor heterogeneity and can be used for disease monitoring during the perioperative course [77]. In a study by Chen and coworkers from 152 HCC patients who underwent liver resection, eight cases with tumor recurrence within two years were selected for single-nucleotide polymorphisms (SNPs) genotyping to investigate the correlation between SNPs of the WWOX gene and the prognosis of HCC patients. The study found that AA + AG genotype and A allele of WWOX rs9926344 were significantly associated with recurrent risk of HCC. Patients carrying rs9926344 AA + AG genotype had poor RFS and OS, suggesting that WWOX rs9926344 polymorphism can be used as an independent prognostic marker for HCC patients after surgery [79]. Therefore, not only liver imaging but genetic sequencing may be used as a tool for postoperative cancer surveillance.

Moreover, resected cancer tissue can be used for developing patient-derived cancer organoids that could overcome the limitations of cancer cell lines and patient-derived xenograft (PDX) models [80]. The possibility to culture tumor and nontumor tissue from the same patient provides the potential for studying tumor development and progression [81].

Broutier and coworkers established cancer organoids derived from HCC patients, confirming that their tumorigenic potential, histological features, and metastatic properties are preserved in vivo [82]. However, the derivation efficiency of HCC organoids was relatively low, with an efficiency rate of about 27% for HCC organoids obtained from surgical resections [82]. This is explained by the fact that cancer organoid cultures could only be established from moderately to poorly differentiated HCCs that typically exhibit a higher proliferative index [82,83].

Another limitation of the cancer organoid cultures is the lack of other cell types apart from epithelial or cancer cells. Stromal, endothelial, and immune cells cannot be expanded simultaneously with cancer organoids using current technology [81].

As already indicated, strict adherence to the BCLC staging system would direct most patients to palliative treatment only. For many years the implementation of the BCLC guidelines respected the ‘stage hierarchy’ approach, where the choice of treatment was exclusively established by the stage of the disease [84]. However, the system’s rigidity limits its utility in real clinical contexts [85,86]. In patients with HCC, prognostic prediction and treatment allocation are complex processes because of the inter-relation of malignant disease, cirrhosis, and additional comorbidities not considered by the BCLC guideline. Another point is that the BCLC approach has a tendency to compromise the use of surgical and loco-regional therapies, and as a consequence, a large proportion of patients in the very early and early stages were treated using palliative therapies [85,86,87]. However, the superiority of liver resection over TACE for patients at the intermediary stage and the superiority of liver resection over the nonsurgical therapies for selected patients at an advanced stage is confirmed in many studies from the eastern world [88,89,90,91]. Another example is patients with portal vein tumor thrombosis who are not candidates for liver resection according to European and American guidelines. However, in the east, an aggressive surgical approach is recommended for certain types of macrovascular invasion [92,93]. The introduction of the ‘treatment stage migration’ and ‘treatment stage alternative’ was an attempt to improve the allocation process, but these two strategies were developed by following the conceptual approach, in which the stage was the dominant driver of therapeutic decisions [84].

**Figure 1:**
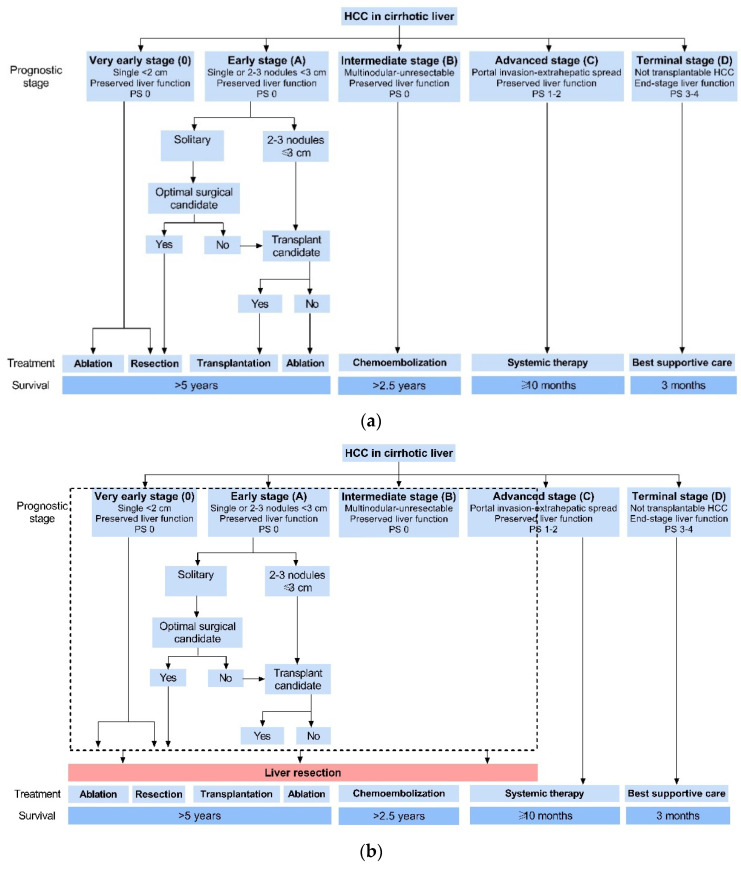
(**a**) Modified Barcelona clinic liver cancer (BCLC) staging and treatment algorithm. (**b**) Real-worldwide clinical practice.

The approach that involves personalized treatment of HCC patients highlighted the importance of a multidisciplinary board discussion aimed at assessing the feasibility of curative-intent therapies in each patient. Thus, the shift is made from the stage-oriented approach to the treatment-oriented approach. The ‘treatment hierarchy’ strategy is based on the hierarchical independence of the treatment choice from HCC staging [84]. An example is the Asia–Pacific treatment algorithm, whereby treatment selection is based on a sequential evaluation of the decision-influencing factors and technical feasibility of liver resection, not limited to a particular staging system [94]. For instance, an international multicenter series published recently indicated that more than 70% of the patients underwent liver resection beyond the BCLC guidelines [95]. Figure 1a shows modified BCLC staging and treatment algorithm, along with (b) real-world clinical practice.

The ‘therapeutic hierarchy’ considers the hierarchical use of a defined scale of treatment modalities established according to survival benefit, in the following order: liver transplantation; liver resection; thermal ablation; intra-arterial therapy and systemic therapy, irrespective of the tumor burden; liver function; and the general conditions of the patient [96].

However, the success of the ‘therapeutic hierarchy’ approach depends on the evaluation of the treatment feasibility by a qualified multidisciplinary board undertaking a decision that extends the selection and access to treatments and improves its outcome [87,97].

The recently published update of the BCLC strategy for prognosis prediction and treatment recommendation (the 2022 update) incorporates two important concepts: (a) ‘treatment stage migration’ (TSM) and (b) untreatable progression [98]. While TSM is applied when a specific patient characteristic requires a shift towards the priority treatment modality for a more advanced stage, untreatable progression relates to the clinical context of the treatment failure or disease progression when patients still fit into their initial stage. However, in this case, the treatment modality considered for a more advanced stage is warranted [98]. Although the recent BCLC update highlights the importance of personalized clinical management, it emphasizes that molecular profiling cannot predict the patient outcome, risk of recurrence after surgery or ablation, or suggest the best treatment option for an individual patient [98,99,100].

Moreover, the latest BCLC update peaks the role of ablation in different clinical scenarios [98]. For very-early-stage patients (BCLC 0), if liver transplantation is not an option, the first recommended treatment modality is ablation as it is associated with similar overall survival to liver resection and significantly fewer complications [101,102]. Ablation is the preferred treatment option for early-stage patients (BCLC A) with a solitary nodule ≤3 cm because it is less invasive and has lower costs and more competitive survival results than those of liver resection [103,104,105]. In patients with up to three nodules each ≤3 cm, it is still debatable whether the outcomes provided by tumor ablation are better than those provided by surgery or even TACE.

## 8. Palliative Treatment Modalities

### 8.1. Precision Medicine and Loco-Regional Procedures

According to BCLC guidelines, transarterial chemoembolization (TACE) and transarterial embolization (TAE) are recommended for intermediate-stage HCC patients to locally control the disease and provide palliative care [1,9]. About 70% of HCC patients at the time of diagnosis have intermediate-to-advanced stage disease, when curative-intent treatment modalities are no longer possible [9,106].

TAE functions by inducing ischemia, resulting in tumor necrosis, whereas in TACE, chemotherapy is applied together with inducing ischemia. Both TAE and TACE provide survival benefits, improving the median survival of HCC patients by up to 26 months [1,9]. However, resistance to these two treatments occurs in approximately 40% of patients [107]. It was already confirmed that performance status, Child–Pugh class, a-fetoprotein level, neutrophil-to-lymphocyte and platelet-to-lymphocyte ratios, and imaging characteristics of HCC response to TACE could predict the outcome [108].

In a recent study by Ziv and coworkers, the impact of precision medicine on TACE performance is determined by assessing the value of genetic and epigenetic factors in predicting HCC response to TACE/TAE [109]. The authors identified molecular subclasses of HCC, nuclear factor E2–related factor 2 (NRF2) pathway-mutated HCC tumors, as being resistant to ischemia, and they could therefore determine the response to TACE/TAE. The study findings indicate that NRF2 pathway-mutated HCC tumors have rapid progression after TAE (6-month cumulative incidence of local progression, 56% vs. 23%; *p* < 0.001), suggesting that these tumors may be resistant to ischemia. It was also shown that ischemia sensitizes NRF2 over-expressing HCC cells to NRF2 inhibition by blocking proliferation (mean doubling time of 1.06 days ± 0.04 in normal and postischemia, and 1.99 days ± 0.23 after short hairpin RNA with ischemia; *p* = 0.01) and increasing cell death (relative potency 160 (range, 62–415) in Huh1 and 175 (range, 65–471) in SNU475 cells after ML385 in ischemia; *p* < 0.001). Importantly, NRF2 inhibition in normal conditions was not lethal to these cells; rather, ischemia induced NRF2 addiction [109]. The authors identified NRF2 pathway mutations in 14% of the study population. The study highlights the fact that NRF2 pathway mutations were not only associated with aggressive tumor features (a larger number of lesions and more aggressive histopathologic characteristics) at baseline but also demonstrated a shorter time to local progression after TAE. The survival and proliferation of NRF2-mutant HCC cell lines depend on NRF2 expression after ischemia. This survival dependency on ischemia-induced NRF2 expression makes these cells particularly sensitive to NRF2 inhibition. Therefore, NRF2 may serve not only as an epigenetic predictor for response to TACE or TAE, but also as a novel therapeutic target [109].

The role of the NRF2 pathway in predicting the response to TACE and TAE was evidenced in a retrospective analysis of a specific subset of patients with HCC who received TACE, suggesting that elevated PKM2 expression is associated with reduced survival [110]. Increased PKM2 expression was demonstrated not only in HCC, but also in many cancer types as an indicator of negative prognosis [111].

The consequence of the study by Ziv and coworkers is that predicting the HCC response to transarterial therapies implies tumor categorization based on molecular characteristics and not solely on imaging characteristics. The use of genetic and epigenetic markers to identify therapeutic vulnerability could change the diagnosis and treatment of HCC, which so far was based on BCLC staging. This would require more pretreatment tissue sampling. However, molecular profiling would allow personalized treatment options for individual patients with HCC.

The latest BCLC update stratifies an intermediary HCC stage into three subgroups of patients based on tumor burden and liver function: (a) patients with well-defined tumor nodules who are candidates for LT; (b) patients with defined tumor burden and preserved portal flow but without the option for LT; and (c) patients with diffuse and extensive tumor liver involvement [98]. According to this stratification, the second subgroup of patients are the best candidates for TACE [112]. Although systemic therapy is the recommended treatment option for the third subgroup, TACE is still feasible, with limited benefit, however, for treating patients [113].

Another loco-regional treatment modality is transarterial radioembolization (TARE). Unlike TACE, where arterial occlusion results in tumor ischemia, TARE is a form of brachytherapy, in which intra-arterially injected radioactive microspheres are sources for internal radiation triggering radiation-induced tumor necrosis [114]. For that purpose, microspheres loaded with radioactive yttrium-90 (Y-90), iodine-131 (131I), or rhenium-188 (188Re) are used [115,116]. Different from TACE, TARE is a two-stage procedure that, apart from delivering radioactive-containing microspheres to the tumor, has to prevent radioactive spread to non-HCC tissue. This is important because procedure-related complications result from excessive irradiation of nontumor tissues [114]. Moreover, TARE is an expensive and sophisticated modality that requires coordinated work of different specialists in interventional radiology, nuclear medicine, radiotherapy, hepatology, medical oncology, and surgery, which makes the procedure less available worldwide [114]. The advantage of TARE is that it can be performed in patients with portal vein thrombosis and in patients who are not candidates for TACE. Based on the results of the Legacy study, the 2022 BCLC update recognized TARE as a treatment modality to be considered in patients with a single HCC ≤8 cm [98,117]. Although several studies reported improved tumor response rate, disease control rate, and fewer side effects, survival rates are similar to that of TACE. [118,119]. The advantage of TARE over TACE was found in downstaging T3 lesions to T2 (58% compared to 31%), suggesting that TARE may be considered either as a ‘bridging’ therapy or main therapy in selected patients with contraindications for TACE [98,120,121].

External beam radiotherapy (EBRT), mainly stereotactic body radiotherapy (SBRT), although not included in the 2022 BCLC update, demonstrated safety and efficacy in treating HCC patients from all BCLC stages [122]. This was confirmed in multiple prospective and randomized controlled trials [123,124,125]. The American Society for Radiation Oncology (ASTRO) published evidence-based practice guidelines recommending the use of EBRT as a potential first-line treatment modality for HCC patients who are not candidates for curative treatment options; as consolidative therapy after an incomplete response to other loco-regional therapies; and as a salvage option for local recurrence [126]. Moreover, NCCN and ESMO guidelines included EBRT as a nonsurgical treatment option for liver-confined HCC [127,128].

### 8.2. Targeted Therapies for HCC

In recent years, extended efforts were spent identifying somatic mutations relative to the development of HCC [129,130]. Most studies, employing next-sequencing technology, have focused on several genes—such as TERT, TP53, CTNNB1, ARID1A, ADRI2, NFE2L2, and KEAP1—with the aim of developing anticancer treatment through targeted therapies [131]. Moreover, several major pathways are mostly aberrant in HCC, including telomere maintenance, TP53/cell cycle, WNT/β-catenin, chromatin remodeling, PI3K/RAS/mTOR pathway, oxidative stress pathways (KEAP1-NRF2 pathway), and angiogenesis [129]. However, for most of the prevalent drivers detected in HCC, targeted therapies are not yet available [132].

Systemic therapy is allocated for advanced-stage HCC when macrovascular invasion or extrahepatic metastases are present [1,9]. Many studies evidenced that HCC is one of the cancers most resistant to cytostatic treatment. Therefore, no systemic therapy was recommended until 2007, when sorafenib was confirmed as an effective treatment for HCC patients at an advanced stage [133]. Since then, the US Food and Drug Administration (FDA) approved several drugs for HCC established as targeted therapy.

## 9. First-Line Therapy

### 9.1. Sorafenib

Sorafenib is an oral multi-TK-receptor inhibitor that suppresses angiogenic vascular endothelial growth factor receptors (VEGFRs), platelet-derived growth factor receptor-β (PDGFRβ), and drivers of cell proliferation (RAF1, BRAF, and KIT) [134].

After the survival benefit for advanced HCC patients was demonstrated in the Phase 2 trial, a large, double-blind, placebo-controlled Phase 3 study was conducted to confirm prolonged survival after sorafenib [133,135]. This trial demonstrated a 31% decrease in relative risk of death (HR 0.69, 95% CI 0.55–0.87; *p* < 0.001) in the sorafenib group compared to that of the placebo group. The median survival was prolonged from 7.9 months in the placebo group to 10.7 months in the sorafenib group. Another Phase 3 study investigated survival outcomes in Asian patients with predominantly HBV-related HCC [136]. The study confirmed a similar benefit concerning the overall survival. However, the adverse events (AEs) rate was 15% in the sorafenib group and 7% in the placebo group.

Sorafenib is documented as a well-tolerated therapy. The most commonly observed drug-related AEs are diarrhea, hand–foot skin reaction, fatigue, and hypertension [133]. The safety and efficacy of sorafenib provided the frame for further clinical research involving patients at earlier HCC stages. In the randomized Phase 3 trial, the time to progression of patients with unresectable HCC managed by a combination of sorafenib and TACE was not prolonged [137]. Similarly, in the adjuvant settings after surgical resection or local ablation, sorafenib did not improve recurrence-free survival [138]. Phase 3 SARAH72 and SIRveNIB73 were designed to compare the survival of patients with advanced HCC between sorafenib and internal radiation with 90Y microspheres [139,140]. The studies showed similar overall survival between the treatment arms, although both studies demonstrated better response rate and quality of life in the radioembolization group.

Indications for sorafenib administration are preserved liver function (Child–Pugh class A) and advanced BCLC stage. Sorafenib is also indicated for HCC patients at the intermediary BCLC stage after disease progression following TACE treatment or in cases where TACE is not technically feasible. Sorafenib treatment should be interrupted if imaging studies confirm the signs of disease progression. Second-line therapy is then recommended.

### 9.2. Lenvatinib

Lenvatinib was introduced by the Tsukuba Research Laboratory in Japan as an angiogenesis-inhibitor [141]. Levantinib is a multi-TK-receptor inhibitor targeting VEGFR, fibroblast growth factor receptors (FGFR), PDGRα, KIT, and RET [2]. The drug was tested in Phase 2 and Phase 3 trials for the treatment of advanced HCC [142,143].

An open-label, Phase 3, multicenter, noninferiority trial was designed primarily to evaluate overall survival after lenvatinib and sorafenib therapy in advanced HCC, and it showed that the median survival for lenvatinib (13.6 months) was noninferior to sorafenib (12.3 months) [143]. In addition, lenvatinib demonstrated higher objective and tumor response rates than sorafenib. In the subgroup analysis of HBV-related HCC, lenvatinib prolonged overall survival for 5 months compared to that of sorafenib, and the objective response rate was also increased. In 2018, lenvatinib was approved as a first-line therapy for advanced HCC by the US FDA based on the previously mentioned results. Lenvatinib and sorafenib were not compared so far regarding cost-effectiveness, and there are no proposed biomarkers to predict drug responses.

The most common registered AEs during lenvatinib administration are hypertension, diarrhea, and loss of appetite with decreased weight [142,143].

Lenvatinib is indicated as an alternative drug to sorafenib in the first-line treatment of advanced-stage HCC (except in cases where more than half of the liver is affected by tumor) or in patients at intermediate HCC stage progressing after TACE treatment.

## 10. Atezolizumab plus Bevacizumab

Atezolizumab is a programmed death 1 (PD-1) inhibitor that selectively targets PD-L1 hindering interaction with receptors PD-1 and B7-1 [144]. Bevacizumab is a monoclonal antibody that targets VEGF, leading to inhibition of angiogenesis and tumor growth [145,146]. 

Although PD-1 inhibitors demonstrated promising clinical results for HCC in Phase 1/2 studies with the response rates ranging from 15% to 20%, improved overall survival was not found in Phase 3 studies [147,148,149,150].

The rationale for the combination of VEGF and PD-1 inhibitors in HCC is related to the effect of impaired VEGF-mediated immunosuppression within the tumor and its microenvironment by VEGF inhibitors, leading to enhanced anti-PD-1 efficacy [151,152]. Moreover, anti-VEGF therapy promotes T-cell infiltration in tumors [153,154].

The clinical benefit of the combination of VEGF and PD-1 inhibitors in HCC was confirmed by the Phase 3, randomized trial IMbrave150 comparing atezolizumab plus bevacizumab over sorafenib [155]. The study enrolled patients with an ECOG 0 or 1 and good liver function (CP class A) [156]. The presence of autoimmune disease and untreated esophageal varices were critical exclusion criteria [155]. Atezolizumab plus bevacizumab was superior to sorafenib regarding mPFS (6.8 vs. 4.3 months, HR: 0.59, *p* < 0.001) and overall survival at 12 months, 67.2% (95% CI, 61.3 to 73.1) vs. 54.6% (95% CI, 45.2 to 64.0) [156]. Regarding safety, 15% of patients had to stop the combination therapy for AEs vs. 10% of patients who were on sorafenib treatment [156]. Upper gastrointestinal bleeding occurred in 6.4% of patients treated by combination therapy [156]. Based on these promising results, NCCN Guidelines Version 5.2020 Hepatocellular Carcinoma recommended atezolizumab plus bevacizumab for the first-line treatment of advanced HCC [156].

## 11. Donafenib

Donafenib is a derivative of sorafenib, and it has the same features as sorafenib: it is an oral, multikinase inhibitor of VEGFR, PDGFRβ, and RAF1 kinases [157].

The clinical benefit of this novel drug was confirmed by a head-to-head, Phase 2–3 study that evaluated the efficacy and safety of first-line donafenib compared with that of sorafenib in 668 Chinese patients with advanced HCC [157]. The primary end point was OS. The study showed that donafenib significantly prolonged OS, 12.1 vs. 10.3 months, respectively (HR, 95% confidence interval, 0.699–0.988; 0.83; *p* = 0.0245) [157]. Moreover, donafenib demonstrated an improved safety and tolerability that, according to the authors, contributed to improved patient adherence. Grade 3 or 4 AEs and common side-effects, such as hand-foot syndrome and diarrhea, occurred less frequently in patients receiving donafenib compared to that of patients on sorafenib (38% vs. 50%; *p* = 0.0018) [157].

From June 2021, donafenib was approved in China to treat unresectable HCC patients who did not receive systemic treatment. However, it is not yet available in the United States.

## 12. Second-Line Therapy

Second-line therapy is intended for advanced HCC patients who experience disease progression after first-line therapy or in cases of drug intolerance.

## 13. Regorafenib

Regorafenib is a multikinase inhibitor with an ultrastructure similar to sorafenib, and it expresses a stronger affinity toward VEGFR [158]. Results from the Phase 3 RESORCE trial indicated better overall survival after regorafenib compared to that of placebo (10.6 vs. 7.8 months) in HCC patients who tolerated and progressed on sorafenib [159]. Moreover, regorafenib was associated with improved objective response rate and progression-free survival. Drug-related AEs were hand-foot skin reactions, diarrhea, and hypertension. Subsequent cost-effectiveness analysis suggested that regorafenib is not cost-effective because of the modest incremental benefit in contrast to high incremental cost [160]. Despite these observations, the FDA and the European Medical Agency approved regorafenib in second-line therapy for patients progressing on sorafenib.

## 14. Cabozantinib

Cabozantinib is a multitargeted TK inhibitor with a specific affinity to the hepatic growth factor receptor MET responsible for HCC carcinogenesis and sorafenib resistance [161]. The randomized Phase 3 trial compared cabozantinib with placebo in previously treated patients with advanced HCC [162]. Treatment with cabozantinib improved OS (8 to 10.2 months) and progression-free survival (1.9 to 5.2 months) compared to that of placebo. High-grade AE (Grade 3 or 4) occurred in 68% of patients in the cabozantinib group and in 36% of the placebo group. During the study, the most common events were palmar-plantar erythrodysesthesia, hypertension, increased aspartate aminotransferase levels, fatigue, and diarrhea. These AEs can be successfully managed by the adopted strategy of supportive care and dose modifications [163]. The comparative cost-effectiveness studies from Germany and the United States found that cabozantinib is not cost-effective compared to that of the best supportive care [164]. Furthermore, cabozantinib was not shown to be cost-effective in almost all scenarios using sensitivity analyses.

## 15. Ramucirumab

Ramucirumab is an anti-VEGFR2 monoclonal antibody that prevents the specific binding between VEGF and VEGFR2 to inhibit angiogenesis [129]. Antitumor activity against advanced HCC was demonstrated for the first time in a Phase 2 pilot study, and mild to moderate AEs were manageable and acceptable [165]. Encouraged by the results of the mentioned study, ramucirumab was tested as second-line therapy in the Phase 3 REACH trial that included advanced-stage HCC patients previously treated by sorafenib [166]. The primary endpoint was negative. Therefore, second-line treatment with ramucirumab did not significantly improve survival over placebo. A subgroup analysis of patients with AFP levels ≥400 ng/mL had a significantly better median overall survival with ramucirumab (7.8 months) than placebo (4.2 months). Thereafter, the randomized Phase 3 trial (REACH-2) demonstrated improved overall survival for ramucirumab compared to that of placebo in patients with HCC and α-fetoprotein concentrations of more than 400 ng/mL who previously received sorafenib [167]. In the Japanese REACH-2 subpopulation, ramucirumab improved progression-free survival and overall survival [168]. The REACH-2 trial indicated that ramucirumab is the first effective agent in the subpopulation of HCC patients selected by biomarker level.

Phase 3 trials confirmed the safety of ramucirumab. Ramucirumab is a well-tolerated drug in the study population. The safety profile of ramucirumab was manageable, including grade 3 or more AEs—hypertension, hyponatremia, and increased aspartate aminotransferase.

## 16. Immune Checkpoint Inhibitors

HCC is one of the inflammation-related carcinomas arising from a microenvironment infiltrated by T cells, natural killer cells, and myeloid cells. Complex interactions between immune cells and tumor cells determine the natural tumor behavior and response to therapy [169]. High immune infiltration, including T-cells (mainly CD8+) and low levels of macrophages, is associated with improved overall survival [170]. Contrarily, immune-suppressive T-cells, dysfunctional NK cells, tumor-associated macrophages, and myeloid-derived suppressor cells are more accumulated in HCC with poor prognosis. Immune checkpoint molecules, including cytotoxic T lymphocyte antigen-4 (CTLA-4), programmed cell death (PD-1), and its two known ligands, PD-L1 and PD-L2, are dominantly expressed on T cells. It is assumed that these molecules have an implication in HCC immune exhaustion [171]. Moreover, the underlying mechanism of immune checkpoint inhibitors is a blockade of negative feedback pathways of the immune system that mediate immunosuppression. The clinical implication of novel therapeutical targets is under investigation. Currently, available drugs are monoclonal antibodies to CTLA-4 (ipilimumab and tremelimumab), monoclonal antibodies to PD-1 (nivolumab, camrelizumab, and pembrolizumab), or PD- L1 (atezolizumab, avelumab, and duravalumab).

## 17. Pembrolizumab

The clinical efficacy of pembrolizumab was evaluated in the Phase 2 KEYNOTE-224 study, which enrolled 104 eligible patients (chronic HBV and HCV with Child–Pugh A liver function and HCC BCLC B or C stage) with confirmed disease progression after or intolerance to sorafenib [147]. The study reported an ORR of 17%; DCR was 62%, and the median DOR was not reached. However, pembrolizumab was a safe and well-tolerated drug with certain antitumor activity. Based on study results, in November 2018, pembrolizumab was granted FDA approval for advanced HCC patients with prior sorafenib treatment. The following Phase 3 KEYNOTE-240 trial was carried out, but the study did not meet its primary endpoint, and the pembrolizumab was not superior to placebo in terms of OS (13.9 vs. 10.6 months, HR = 0.78; *p* = 0.02) and PFS (3 vs. 2.8 months; HR = 0.77; *p* = 0.01) [149]. However, the results were consistent with those of KEYNOTE-224: the ORR was 18.3%, and DCR was 62,2%, while median OS was longer in the pembrolizumab arm compared to that of placebo, supporting a favorable risk-to-benefit ratio for pembrolizumab. No hepatitis B or C flares were identified [149]. In 2021, the FDA approved pembrolizumab for HCC patients not eligible for bevacizumab (front-line allocation to atezolizumab plus bevacizumab). In the meantime, the results from the ongoing Phase 3 KEYNOTE-394 trial in Asian patients are awaited.

## 18. Camrelizumab

Camrelizumab is a programmed cell death 1 (PD-1) inhibitor that was recently investigated as a treatment for various malignancies, including B cell lymphoma, Hodgkin lymphoma, HCC, gastric and gastroesophageal cancers, and nonsmall cell lung cancer. A multicenter study performed on Chinese patients with advanced HCC confirmed the antitumor activity of camrelizumab [172]. This open-label, randomized, Phase 2 trial included 220 eligible patients with advanced HCC with progression or intolerance to previous treatments. The objective response was reported in 32 of 217 patients (14.7%; 95% CI 10.3–20.2), and the overall survival probability at 6 months was 74.4% (95% CI 68.0–79.7) [172]. The authors concluded that camrelizumab might represent a new treatment option for patients with advanced HCC who had progression on previous treatment modalities.

## 19. Nivolumab

Nivolumab is a monoclonal antibody to PD-1 that was recently introduced in HCC targeted therapy. An open-label, noncomparative, Phase 1/2 dose escalation and expansion trial (CheckMate 040) demonstrated an ORR of 20%, with 3 complete responses and 39 partial responses [148]. The most common AEs were fatigue, musculoskeletal pain, pruritus and rash, and diarrhea, and only 11% of patients had to discontinue the treatment because of the AEs’ occurrence [148]. The FDA granted accelerated approval to nivolumab for patients with advanced-stage HCC after the failure of sorafenib. Moreover, approval for the use of nivolumab, pembrolizumab, and other immune checkpoint inhibitors changed the current HCC treatment strategy and significantly extended an OS and PFS in a large proportion of patients.

Various combinations with biological therapy or dual immune checkpoint blockade were assessed either for the first- or second-line therapy. After the preliminary results of Phase 1/2 studies, a comparative randomized controlled trial (HIMALAYA study) was designed to compare Duravalumab versus the combination of duravalumab plus tremelimumab versus sorafenib [173]. The results are still pending. The efficacy of the combination of immune checkpoint inhibitors with bevacizumab on the long-term survival of patients with unresectable HCC was demonstrated in the two randomized controlled trials [155,156].

**Figure 2:**
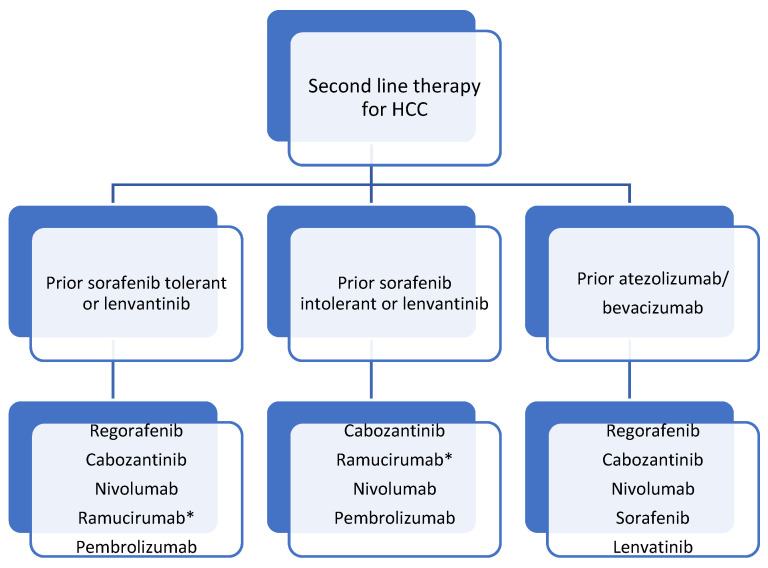
Second-line treatment options for advanced hepatocellular carcinoma. HCC—hepatocellular carcinoma. * Only in patients with AFP > 400 ng/mL.

As previously mentioned, the efficacy of cellular immunotherapy for advanced HCC after different interventional therapeutic strategies significantly changed over the past few years, showing that a second-line regimen mainly depends on the previous treatment used (Figure 2 shows proposed second-line regimens after the previous first-line therapy used).

Combination immunotherapy, including dendritic cells and cytokine-induced killer cells with routine treatments for HCC, was shown to improve patients’ prognoses. Moreover, immunotherapy, including cytokine-induced killer cellular therapy, could increase OS and decrease recurrence rate after surgical resection, local tumor ablation, and TACE. According to a recent meta-analysis, immunotherapy brought new hope into this field, and nowadays, it has a favorable role in improving early or long-term efficacy for HCC and is well accepted with a proven survival benefit [174,175].

## 20. Conclusions

Today, hepatocellular carcinoma (HCC) is one of the rare cancers characterized by unrestrainedly growing incidence. In some countries, it is the fastest-rising cause of cancer-related deaths. Despite apparent improvements in surveillance programs, diagnostic and treatment procedures, molecular therapies, and numerous research initiatives, it remains an uncontrollable disease. The successful implementation of HCC-preventive strategies, including screening and chemopreventive intervention, requires an individual, risk-based, and tailored approach. The potential impact of precision medicine on the outcome of HCC treatment should cope with the resistance to performing pretreatment biopsies. The use of next-generation sequencing—providing multicell type characterization of diseased liver tissue microenvironment at risk of cancer—may facilitate the development of effective chemoprevention strategies. However, the tailored approach should provide cost-effective and precise preventive intervention in the clinical management of patients at risk of HCC. Identification of biomarkers with high sensitivity and specificity is important for the development of personalized treatment plans. Postoperative monitoring also relies on the exploration of HCC biomarkers. In the future, studies investigating genetic and epigenetic predictors of tumor response will be needed, as they can significantly affect the outcomes of patients with HCC. In daily clinical practice, the shift from the ‘stage hierarchy’ to ‘therapeutic hierarchy’ is critical to systematically directing the choice of HCC treatment toward the potentially most effective option on an individual basis. In that context, precision medicine promotes patient management based on an individual approach rather than on the basis of disease stage, in the knowledge that the final decision must be approved by a multidisciplinary expert team. In the future, studies on the liver microenvironment and a better understanding of its heterogeneity are needed to improve HCC treatment efficacy.

## Figures and Tables

**Table 1:** Comparison between liver biopsy and liquid biopsy.

	Target	Advantages	Disadvantages
Liver biopsy	Histology Histopathology Immunohistochemistry	Inexpensive Gross pathology and growth pattern assessment Assessment of microvascular invasion Prognostic stratification Revision by other pathologists	Technical challenges during lesion targeting Intervention-related complications Needle track seeding Inadequate or failed sampling
Liquid biopsy	Circulating tumor cells Circulating tumor DNA Cell-free microRNA Extracellular RNA	Determination of molecular subtype Noninvasiveness Monitoring of tumor recurrence, progression, and therapeutic response in real time Overcome heterogeneous tumor biology	Nonstandardized techniques of sampling Different techniques for detection Multiple marker analysis Need for validation in clinical settings in the future Expensive Lower sensitivity and precision
